# Good Health and Wellness: Measuring Impact Through an Indigenous Lens

**DOI:** 10.5888/pcd16.180655

**Published:** 2019-08-15

**Authors:** Thomas J. Lawrence, Rosalina D. James

**Affiliations:** 1 Seattle Indian Health Board, Urban Indian Health Institute, Seattle, Washington

## Abstract

In 2014, the Centers for Disease Control and Prevention (CDC) commissioned the Urban Indian Health Institute (UIHI) to coordinate a multifaceted national evaluation plan for Good Health and Wellness in Indian Country (GHWIC), CDC’s largest investment in chronic disease prevention for American Indians and Alaska Natives (AI/ANs). GHWIC is a collaborative agreement among UIHI, CDC, tribal organizations, and individual tribes. In collaboration, UIHI and CDC drew upon an indigenous framework, prioritizing strength-based approaches for documenting program activities, to develop a 3-tiered evaluation model. The model incorporated locally tailored metrics, adherence to tribal protocols, and cultural priorities. Ultimately, federal requirements and data collection processes were aligned with tribal strengths and bidirectional learning was promoted. We describe how UIHI worked with tribal recipients, tribal health organizations, Tribal Epidemiology Centers, and CDC to develop and implement the model on the basis of an indigenous framework of mutual trust and respect.

**Summary**

**What is already known on this topic?**

American Indians and Alaska Natives suffer disproportionate health disparities and chronic disease rates compared with other populations. To address disparities, a history of mistrust between tribal communities and the federal government regarding evaluation and other data-driven practices must be confronted.

**What is added by this report?**

We describe an Indigenous Evaluation Framework, emphasizing indigenous core values and knowledge, used to evaluate a federally sponsored initiative to prevent chronic disease in Indian Country.

**What are the implications for public health practice?**

Our work improves public health practice by reinforcing how indigenous ways of gathering knowledge are as valid and effective as Western methods.

## Introduction

The Centers for Disease Control and Prevention (CDC) launched Good Health and Wellness in Indian Country (GHWIC) in 2014 to reduce American Indian and Alaska Native (AI/AN) health disparities, including commercial tobacco use, obesity, and disability and premature death as a result of diabetes, heart disease, and stroke. GHWIC sought to build an overall evaluation plan by using an indigenous framework to match the locally tailored, culturally driven program approach. Historically, research and evaluation efforts have abused and exploited AI/AN communities, such as in the 1979 Barrow alcohol study that published negative reports about Inupiaq villages without consulting those communities (1). Pervasive mistrust of these practices has limited AI/AN partnerships with federal agencies (2), explaining why AI/AN communities are reluctant to collaborate with external organizations that claim they want to help improve tribal health (1). Joan LaFrance states in *Reframing Evaluation: Defining an Indigenous Evaluation Framework*, “The field of evaluation draws heavily on research methodologies that can be considered invasive when imposed by outside funding agencies” (3). CDC collaborated with tribal communities by using an indigenous approach to create a 3-tiered evaluation model that combines indigenous knowledge and values with Western evaluation practices.

## Purpose and Objectives

The overall purpose of GHWIC was to create an AI/AN-owned and AI/AN-centered public health initiative designed to meet AI/AN needs, allowing recipients to take charge of their communities’ health while achieving chronic disease prevention and health promotion outcomes. Within this purpose, 4 evaluation objectives were identified: 1) share successes and lessons learned across Indian Country and beyond, 2) report outcome data to GHWIC communities for feedback and guidance on program implementation and improvement, 3) expand the evidence base for chronic disease prevention in Indian Country, and 4) support opportunities for future funding to promote AI/AN community health programs.

GHWIC recipients include 12 federally recognized tribes and 11 tribal health organizations (THOs) that serve tribes in their Indian Health Service (IHS) service regions. The program operates at 3 interconnected levels: local communities (tribes), regions (typically, THOs or Tribal Epidemiology Centers [TECs] serving all or most tribes in their regions), and nationally (Indian Country-wide). The evaluation approach mirrors this program design with the Urban Indian Health Institute (UIHI) focusing on all AI/AN communities served and supporting all GHWIC recipients. CDC supports GHWIC recipients and provides data on GHWIC national outcomes, accomplishments, challenges, and progress.

## Intervention Approach

The framework for the GHWIC initiative and evaluation approach began to take shape as early as 2011 when CDC’s tribal advisory committee — a federally mandated committee to advise CDC on tribal matters — encouraged CDC’s National Center for Chronic Disease Prevention and Health Promotion (NCCDPHP) to more thoughtfully address serious health disparities among AI/ANs while respecting and incorporating indigenous knowledge. As a result, NCCDPHP staff participated in the first CDC listening sessions with tribal health leaders and visited tribes and tribal organizations over several years before launching the GHWIC initiative. In these sessions and visits, tribal partners offered knowledge and wisdom to CDC staff on how federal funding does not take the AI/AN perspective into account and how tribes and CDC could work together. These activities helped to expand the Western approach to understanding health and disease and how to track progress toward improving health by integrating cultural values of indigenous communities. A key discussion point was the importance of identifying principles of program development and evaluation processes with the people and organizations served by CDC funds. CDC’s understanding continued to evolve during the GHWIC program period, from 2014 through 2019, as recipients and CDC struggled with conflicting demands of meeting AI/AN cultural imperatives and required deliverables. Through collaboration, listening, and learning, GHWIC was eventually able to develop culturally sound programs and evaluation approaches tailored to specific indigenous cultural customs and tribal protocols.

The Indigenous Evaluation Framework describes the following 4 core values that were adapted by the GHWIC model (Table) (3):

**Table:** Core Indigenous Values That Guided the Good Health and Wellness in Indian Country (GWHIC) Project^a^

Core Indigenous Values	Definition	Indigenous Evaluation Examples
**Centrality of the community and family**	Engage the community when planning and implementing evaluation, making evaluation processes transparent. Understand that programs may focus on restoring community health and wellness and individual achievements.	• CDC participated in tribal listening sessions and used findings to shape GHWIC initiative. • CDC engaged GHWIC recipients in the evaluation development, planning, and implementation.
**People of place**	Recognize a tribal entity’s relationship to its land, history, and historical events in relation to current health conditions and individuals affected. Respect and avoid generalizations among tribal entities, understanding that what occurs in one place may not translate to other situations or other places.	• CDC respected that all tribes and tribal organizations are unique. • Performance measure selection was flexible. • All are not the same, what works in one place might not work in another. • Programs were created in relationship to a specific community, its history, and its current situation.
**Recognizing individual gifts**	Use a holistic approach to evaluate while acknowledging that there are different ways of conducting evaluation.	• CDC respected that individual tribal chronic disease prevention programs operated according to local cultural context. • Tribal data are not always quantifiable, thus stories and storytelling (qualitative evaluation) are just as important and effective. • CDC, UIHI, tribes, and tribal health organizations offered multiple venues to showcase successes and learning opportunities such as UIHI story map, http://www.uihi.org/projects/good-health-wellness-in-indian-country/.
**Personal and tribal sovereignty**	Embody respect for tribal approval processes that build greater capacity in communities, and report findings in ways that are meaningful and impactful.	• All data, photos, stories and reports may be used only with proper tribal permissions and approvals by the tribes or tribal health organizations (see http://www.uihi.org/projects/good-health-wellness-in-indian-country/ for GHWIC reports) • Recipient feedback is solicited on evaluation reports and materials. • Tribes and tribal health organizations are consulted in the development of reports, ensuring reports are meaningful to tribal audiences and federal funders. • Communication was iterative, and approval was obtained from recipients regarding products before dissemination.

Abbreviations: CDC, Centers for Disease Control and Prevention; UIHI, Urban Indian Health Institute.

a Indigenous values and definitions from LaFrance (3).

1. **Centrality of the Community and Family** requires engaging the community when planning and implementing an evaluation, making evaluation processes more transparent, and highlighting the importance of community health in addition to individual achievements.
2. **People of Place** recognizes a tribal entity’s relationship to land and history, how historical events have shaped current health conditions, and the uniqueness of place and history. What occurs in one place might not be generalizable to other situations or other locations.
3. **Recognizing Individual Gifts** prompts evaluators to take a holistic approach to evaluate while acknowledging that there are different ways of conducting evaluation.
4. **Upholding Personal and Tribal Sovereignty** embodies respect for tribal approval processes, building greater capacity within recipient communities and reporting findings in ways that are meaningful and impactful to recipients.

Integrating these values required federal partners and public health professionals to recognize the importance of culture in AI/AN health. Cultural centrality and mutual respect were critical to effectively implement the GHWIC initiative and its evaluation. The indigenous framework emphasizes the criticality of local context and community knowledge in documenting program gaps and successes. To accomplish federal goals while aligning data processes with tribal recipient values, GHWIC established a systematic approach to promote bidirectional learning for both program and evaluation implementation. Technical assistance (TA) was provided to recipients through the Evaluation TA Exchange Partners (EEPs) model. The EEPs model involved members of the GHWIC evaluation team, which included CDC and UIHI staff, to provide ongoing, one-on-one support to recipients throughout the cooperative agreement. EEPs were matched with recipients, based on outcomes, geography, and staffing capacity and worked with recipients through video conferences, site visits, and ad hoc communications to share guidance on evaluation plans, performance measurements, and annual report development.

Indigenous methods coupled with community-based participatory approaches emphasize transparency, equitable partnerships, and actionable objectives to promote sustainable change for improved health outcomes. Evaluation efforts that include community participation in the design, development, and implementation are more likely to effectively document long-term change and program gaps (3). UIHI and CDC integrated indigenous values and CDC priorities into the evaluation plan to create an approach that met the needs of diverse recipients and partners to assess impact and identify successful strategies for chronic disease prevention in tribal settings.

## Evaluation Methods

The 3-tiered model (Figure) was developed as a guide to capture GHWIC progress and impact on improving tribal health and wellness. Evaluation questions were designed in close partnership with CDC and UIHI to reflect activities at the local level, with tribes and THOs collecting local evaluation data (Tier 1). TECs collaborate with tribes and THOs within each IHS region to provide technical assistance and collect quantitative and qualitative data (Tier 2). UIHI reported aggregated data of all GHWIC recipients to CDC, while CDC reported aggregated data to its leadership and federal decision makers (Tier 3). At the local level, recipients tracked site-specific progress over time, including process and summative outcomes; TECs collected and assembled information from primary data collection and public databases. UIHI serves as the national coordinating center for evaluation across all partners, compiling and summarizing data, and ensuring the GHWIC evaluation honored core indigenous values and cultures. UIHI facilitated key data collection and management practices, documenting processes and outcomes as they aligned with tribal chronic disease prevention efforts.

**Figure:**
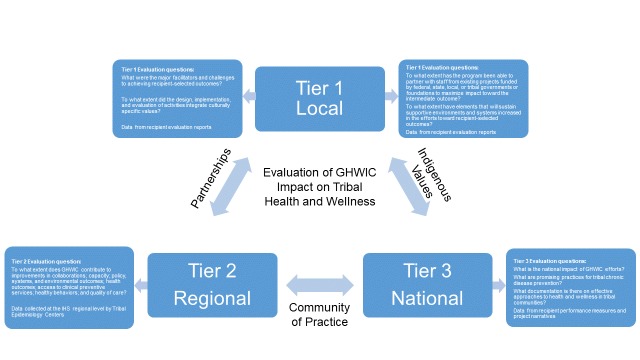
Conceptual diagram of the 3-tiered evaluation of Good Health and Wellness in Indian Country (GHWIC) impact on tribal health and wellness, with questions that are answered within each tier. Abbreviation: IHS, Indian Health Service. Figure was created by the Urban Indian Health Institute.

## Results

In the early stages of the GHWIC initiative, UIHI, CDC evaluation staff, and recipient representatives identified a series of performance measures and evaluation outcomes that would contextualize GHWIC efforts, identify successful activities, and provide lessons learned for others supporting similar tribal health promotion programs. With these metrics, UIHI and CDC compiled findings and created reports documenting the regional and national impact of GHWIC. For example, aggregated data show that from 2014 through 2017, approximately 15,000 AI/AN people had better access to healthier foods through 16 new tribal settings with low sodium nutrition guidelines and 77 new tribal settings promoting nutritious foods (4). GHWIC recipients also built healthier and more active communities. More than 14,500 AI/AN people improved access to physical activity with 91 new policies promoting physical activity (5).

## Implications for Public Health

The GHWIC indigenous framework placed recipient (Tier 1) knowledge at the forefront of developing effective and sustainable health interventions in tribal communities for a true reflection of the impact of those interventions. Implementing the 3-tiered evaluation model offered UIHI the ability to measure impact through an indigenous lens with collaborative decision making and bidirectional learning among tribal and federal partners. At the local level, recipients established evaluation indicators that best reflected the qualities of their cultural and community landscapes. At the regional level, THOs and TECs developed strong partnerships and trust with tribes in their regions, allowing them to collect and analyze local and regional data. Data from these 11 regions were then combined to assess national impact. However, the GHWIC evaluation had limitations. Because of the flexibility in performance measure selection, methods for data collection varied, making aggregate data assessment inclusive of all recipient activities difficult. In addition, not all tribal communities in the United States were represented in the GHWIC initiative. Despite these shortfalls, the GHWIC evaluation model provided an important framework for a tribal-CDC partnership to achieve increased AI/AN health and wellness.
